# Exosome-delivered microRNAs of “chromosome 19 microRNA cluster” as immunomodulators in pregnancy and tumorigenesis

**DOI:** 10.1186/1755-8166-5-27

**Published:** 2012-07-02

**Authors:** Jörn Bullerdiek, Inga Flor

**Affiliations:** 1 Center for Human Genetics, University of Bremen, Leobener Str. ZHG, Bremen, 28359, Germany

**Keywords:** MicroRNA, Chromosomal translocation, Thyroid adenoma, C19MC, Placenta, Epigenetics, Immunomodulation

## Abstract

**Background:**

Structural rearrangements of chromosomal band 19q13 are a non-random cytogenetic abnormality in thyroid adenomas and adenomatous goiters and lead to an expression of miRNAs of the chromosome 19 microRNA cluster C19MC. Normally, expression of these miRNAs is silenced except for embryonic stem cells and the placenta where they represent the majority of miRNAs not only in the trophoblast but also in exosomes derived from it.

**Presentation of the hypothesis:**

We have advanced the hypothesis that as part of the feto-maternal communication miRNAs of C19MC serve immunomodulatory functions in the placenta and confer a growth advantage to thyroid nodules by protecting them against autoimmune attacks. More precisely, the exosomes containing these miRNAs may specifically target immune cells in their local environment as well as systemically by transferring their cargo to recipient cells. Within these target cells the transferred miRNAs can interact with mRNAs of the recipient cells thereby suppressing their immune-specific functions.

**Testing the hypothesis:**

Experiments used to demonstrate the immunomodulatory capacity of placenta-derived exosomes can be modified by transfecting the target cells with those miRNAs of C19MC represented in placental exosomes.

**Implications of the hypothesis:**

Mimics of C19MC-derived miRNAs might develop to useful drug candidates for the treatment of autoimmune disease as e.g. rheumatoid arthritis and Sjögren’s syndrome and for the prevention of transplant rejection. In case of tumor entities with elevated expression of C19MC miRNAs these miRNAs may be interesting targets for treatment with appropriate antagonists.

## Background

**Figure 1:**
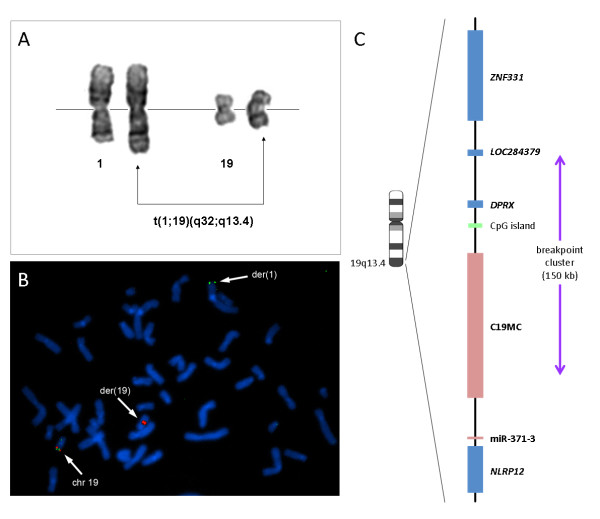
**(A) Partial karyotype of an adenoma with an apparently balanced translocation t(1;19)(q32;q13.4) is shown as an example of adenomas with a 19q13.4 chromosomal rearrangement.** (B) Metaphase of a thyroid adenoma with a t(1;19)(q32;q13.4) after FISH with dual-color break-apart rearrangement probe appropriate for the detection of 19q13.4 rearrangements. The 19q13.4 rearrangement is indicated by one single green and one single red signal each. (C) The chromosomal region 19q13.4 with the miRNA clusters C19MC and miR-371-3 (pink boxes) and surrounding protein-coding genes (blue boxes). The common breakpoint cluster of thyroid adenomas and adenomatous goiters is indicated by an arrow.

First being described in 1989 [1], structural rearrangements of chromosomal band 19q13 are a frequent non-random cytogenetic abnormality in thyroid adenomas and adenomatous goiters. By applying molecular-cytogenetic methods on established cell lines it was possible to narrow down the breakpoints to a region of about 150 kbp [2] which was later shown to harbor C19MC (chromosome 19 microRNA cluster), the largest human microRNA cluster at all [3] (Figure 1). As a rule, by the chromosomal rearrangements the microRNAs of this cluster and the neighboring miR-371-3 cluster become strongly upregulated [4]. Of these both clusters C19MC is remarkable not only because of its sheer size encoding more than 50 mature microRNAs but also because of its “young” age. The whole cluster is primate-specific [3] and thus must have evolved within a relatively short time in terms of evolution. Normally, the miRNAs of C19MC are expressed almost exclusively in embryonic stem cells [5-9] and, later during embryonic and fetal development, only in the placenta [3,10]. Luo et al. [11] demonstrated that the trophoblast secretes exosomes, i.e. small membrane microvesicles, which contain placenta-specific miRNAs. Moreover, it was demonstrated quite recently that the vast majority of miRNAs packed into placenta-derived exosomes consist of miRNAs of the C19MC cluster [12]. However, in both cases, i.e. in the placenta or after chromosomal translocations in benign thyroid nodules, the exact mechanisms by which C19MC miRNAs contribute to normal or aberrant functions, respectively, remain obscure.

Herein, we shall advance the hypothesis that as part of the feto-maternal communication miRNAs of C19MC serve immunomodulatory functions in the placenta and, once re-expressed due to chromosomal translocations, confer a growth advantage to thyroid nodules by protecting them against autoimmune attacks.

## Presentation of the hypothesis

Though cytogenetic aberrations of 19q13.4 as detected in benign thyroid lesions represent one of the most common specific chromosomal alterations in epithelial tumors at all the molecular mechanisms resulting from these frequent genomic alterations still remain obscure. As to tumorigenesis, the stimulation of invasive growth has been attributed to some miRNAs of C19MC [for review see [13] but the activation of the cluster in thyroid adenomas and nodular goiters with 19q13.4 alterations apparently does not coincide with invasive growth. Thus, it seems unlikely that in general activation of C19MC leads to invasive growth or other features characterizing malignant cells. On the other hand, presumed “physiological functions” of its miRNAs might give us a clue to understand their role in tumorigenesis. Of note, until birth expression of C19MC persists only in the placenta or, more precisely, its trophoblast [10,11], and is expressed exclusively from the paternal allele whereas the maternal allele is silenced by epigenetic modification [14].

In general, miRNAs do not necessarily exert their main functions in the cells they are expressed in since considerable amounts of miRNAs can become packed into microvesicles called exosomes. Exosomes are bioactive vesicles derived from endosomal membranes and involved in intercellular communication by their specific cargos of proteins, mRNAs, and miRNAs [for review see [15]. In previous reports placenta-derived exosomes have been demonstrated to interact with immune cells, e.g. resulting in suppression of T-cell signaling components [16,17].

Donker et al. [12] were able to demonstrate recently that the cellular miRNA composition of human primary trophoblast cells strongly resembled that of the exosomes secreted from these cells. In both cases those of C19MC represented the majority of mature miRNAs. Of note, six microRNAs of C19MC ranged among the top-ten exosomal miRNAs. Based on their findings the authors have assumed that these miRNAs “may play an important role in placental-maternal communication, possibly directing maternal adaptation to pregnancy.”

Herein, we would like to outline the hypothesis that as one major function miRNAs of C19MC prevent the embryo from being attacked by the maternal immune system. Immunologically, the embryo is considered being a semi-allograft and in case of egg donation even a full allograft [18]. Nevertheless, the embryo efficiently avoids rejection by its mother’s immune system by mechanisms that are not fully understood yet [for review see [19]. Exosomes are known to share membrane characteristics with the cells they are derived from [20]. Thus, it seems tempting to assume that they act like decoy-flairs for a jet. The exosomes can specifically target immune cells in their local environment, i.e. the decidua, as well as systemically thereby transferring their cargo when melting with the membrane of recipient cells. Within these target cells the transferred miRNAs can interact with mRNAs of the recipient cells thereby modulating post-transcriptional regulation. Non-specific systemic side effects of this mechanism may be the mild immunosuppression noted during pregnancy e.g. leading to the improvement of rheumatoid arthritis [for review see [21].

Tracing back to nodular goiters and thyroid adenomas re-expression of C19MC may protect cells against autoimmune attacks. Of note, a considerable percentage of these lesions develop after a pre-existing autoimmune disease of the thyroid.

Finally, the question arises if malignant tumors can adopt this mechanism to protect themselves. 19q13 is one of the most frequent chromosomal breakpoints identified in human tumors and even if, in particular in case of complex karyotypic aberrations, the small size of chromosome 19 may have resulted in false positive identification of this breakpoint there remain a number of tumor entities where its involvement has been identified unambiguously as e.g. hamartoma of the liver [22,23]. Also, a number of recent papers point to the role of amplification (e. g. in CNS-PNET [24] and in embryonal brain tumors with ependymoblastic multilayered rosettes [25]) or undermethylation of the C19MC locus (e. g. in hepatocellular carcinomas [26,27]) in several tumor entities.

## Testing the hypothesis

There are straightforward appropriate experimental approaches to test our hypothesis. Nevertheless, these approaches are time consuming because not all miRNAs of the cluster may have the capacity to modulate immune cells and because different types of immune cells have to be tested. As a first step the same experiments used to demonstrate the immunomodulatory capacity of placenta-derived exosomes [e.g. [17] can be modified by transfecting the target cells with those miRNAs of C19MC highly represented in placental exosomes. Furthermore, it did not escape our attention that mesenchymal cells from the amniotic membrane have strong immunomodulatory properties, e.g. by actively suppressing T-cell proliferation induced by alloantigens [28]. So far, it is believed that at term only the trophoblast expresses C19MC miRNAs but if our hypothesis holds true one would expect that these amniotic-membrane derived cells do so as well.

## Implications of the hypothesis

If the hypothesis holds true it will be not only relevant in terms of basic science but also for several clinical approaches. Mimics of C19MC-derived miRNAs, either encapsulated or not, might develop to useful drug candidates for the treatment of autoimmune disease as e.g. rheumatoid arthritis and Sjögren’s syndrome. Likewise, these miRNAs may prolong the maintenance of functional allografts. On the other hand, in case of tumor entities with forced expression of C19MC miRNAs their antagonists may represent interesting alternatives for targeted treatments.

## Abbreviations

C19MC, chromosome 19 microRNA cluster; CNS-PNET, Central nervous system primitive neuroectodermal tumor; miRNA, microRNA.

## Competing interests

The authors declare competing financial interests because the University of Bremen is currently applying for a patent claiming the use of C19MC miRNAs for immunomodulation.

## Authors' contributions

Both authors have equally contributed to this hypothesis and written the manuscript. Both authors read and approved the final manuscript.

## References

[B1] Bartnitzke S Herrmann ME Lobeck H Zuschneid W Neuhaus P Bullerdiek J Cytogenetic findings on eight follicular thyroid adenomas including one with a t(10;19) Canc Genet Cytogenet 1989 39 65 68 10.1016/0165-4608(89)90230-6 2731149

[B2] Belge G Rippe V Meiboom M Drieschner N Garcia E Bullerdiek J Delineation of a 150-kb breakpoint cluster in benign thyroid tumors with 19q13.4 aberrations Cytogenet Cell Genet 2001 93 48 51 10.1159/000056947 11474178

[B3] Bentwich I Avniel A Karov Y Aharonov R Gilad S Barad O Barzilai A Einat P Einav U Meiri E Identification of hundreds of conserved and nonconserved human microRNAs Nat Genet 2005 37 766 770 10.1038/ng1590 15965474

[B4] Rippe V Dittberner L Lorenz VN Drieschner N Nimzyk R Sendt W Junker K Belge G Bullerdiek J The two stem cell microRNA gene clusters C19MC and miR-371-3 are activated by specific chromosomal rearrangements in a subgroup of thyroid adenomas PLoS One 2010 5 e9485 10.1371/journal.pone.0009485 20209130 PMC2831057

[B5] Bar M Wyman SK Fritz BR Qi J Garg KS Parkin RK Kroh EM Bendoraite A Mitchell PS Nelson AM MicroRNA discovery and profiling in human embryonic stem cells by deep sequencing of small RNA libraries Stem Cell 2008 26 2496 2505 10.1634/stemcells.2008-0356 PMC2847579 18583537

[B6] Laurent LC Chen J Ulitsky I Mueller FJ Lu C Shamir R Fan JB Loring JF Comprehensive microRNA profiling reveals a unique human embryonic stem cell signature dominated by a single seed sequence Stem Cell 2008 26 1506 1516 10.1634/stemcells.2007-1081 18403753

[B7] Morin RD O'Connor MD Griffith M Kuchenbauer F Delaney A Prabhu AL Zhao Y McDonald H Zeng T Hirst M Application of massively parallel sequencing to microRNA profiling and discovery in human embryonic stem cells Genome Res 2008 18 610 621 10.1101/gr.7179508 18285502 PMC2279248

[B8] Ren J Jin P Wang E Marincola FM Stroncek DF MicroRNA and gene expression patterns in the differentiation of human embryonic stem cells J Transl Med 2009 7 20 10.1186/1479-5876-7-20 19309508 PMC2669448

[B9] Cao H Yang CS Rana TM Evolutionary emergence of microRNAs in human embryonic stem cells PLoS One 2008 3 e2820 10.1371/journal.pone.0002820 18665260 PMC2474702

[B10] Zhang R Wang YQ Su B Molecular evolution of a primate-specific microRNA family Mol Biol Evol 2008 25 1493 1502 10.1093/molbev/msn094 18417486

[B11] Luo SS Ishibashi O Ishikawa G Ishikawa T Katayama A Mishima T Takizawa T Shigihara T Goto T Izumi A Human villous trophoblasts express and secrete placenta-specific microRNAs into maternal circulation via exosomes Biol Reprod 2009 81 717 729 10.1095/biolreprod.108.075481 19494253

[B12] Donker RB Mouillet JF Chu T Hubel CA Stolz DB Morelli AE Sadovsky Y The expression profile of C19MC microRNAs in primary human trophoblast cells and exosomes Mol Human Reprod 2012 “Accepted Article”10.1093/ molehr/gas013 10.1093/molehr/gas013 PMC3389496 22383544

[B13] Flor I Bullerdiek J The dark side of a success story: microRNAs of the C19MC cluster in human tumours J Pathol 2012 “Accepted Article” 10.1002/path.4014 22374805

[B14] Noguer-Dance M Abu-Amero S Al-Khtib M Lefevre A Coullin P Moore GE Cavaille J The primate-specific microRNA gene cluster (C19MC) is imprinted in the placenta Hum Mol Genet 2010 19 3566 3582 10.1093/hmg/ddq272 20610438

[B15] Pant S Hilton H Burczynski ME The multifaceted exosome: Biogenesis, role in normal and aberrant cellular function, and frontiers for pharmacological and biomarker opportunities Biochem Pharmacol 2012 83 1484 1494 10.1016/j.bcp.2011.12.037 22230477 PMC7110994

[B16] Taylor DD Akyol S Gercel-Taylor C Pregnancy-associated exosomes and their modulation of T cell signaling J Immunol 2006 176 1534 1542 16424182 10.4049/jimmunol.176.3.1534

[B17] Sabapatha A Gercel-Taylor C Taylor DD Specific isolation of placenta-derived exosomes from the circulation of pregnant women and their immunoregulatory consequences Am J Reprod Immunol 2006 56 345 355 10.1111/j.1600-0897.2006.00435.x 17076679

[B18] van der Hoorn ML Scherjon SA Claas FH Egg donation pregnancy as an immunological model for solid organ transplantation Transplant Immunol 2011 25 89 95 10.1016/j.trim.2011.06.004 21708252

[B19] Warning JC McCracken SA Morris JM A balancing act: mechanisms by which the fetus avoids rejection by the maternal immune system Reproduction 2011 141 715 724 10.1530/REP-10-0360 21389077

[B20] Mincheva-Nilsson L Baranov V The role of placental exosomes in reproduction Am J Reprod Immunol 2010 63 520 533 10.1111/j.1600-0897.2010.00822.x 20331583

[B21] Ostensen M Villiger PM Forger F Interaction of pregnancy and autoimmune rheumatic disease Autoimmun Rev 2012 11 A437 A446 10.1016/j.autrev.2011.11.013 22154710

[B22] Speleman F De Telder V De Potter KR Dal Cin P Van Daele S Benoit Y Leroy JG Van den Berghe H Cytogenetic analysis of a mesenchymal hamartoma of the liver Canc Genet Cytogenet 1989 40 29 32 10.1016/0165-4608(89)90142-8 2758398

[B23] Rajaram V Knezevich S Bove KE Perry A Pfeifer JD DNA sequence of the translocation breakpoints in undifferentiated embryonal sarcoma arising in mesenchymal hamartoma of the liver harboring the t(11;19)(q11;q13.4) translocation Genes Chromosomes Canc 2007 46 508 513 10.1002/gcc.20437 17311249

[B24] Li M Lee KF Lu Y Clarke I Shih D Eberhart C Collins VP Van Meter T Picard D Zhou L Frequent amplification of a chr19q13.41 microRNA polycistron in aggressive primitive neuroectodermal brain tumors Canc Cell 2009 16 533 546 10.1016/j.ccr.2009.10.025 PMC3431561 19962671

[B25] Nobusawa S Yokoo H Hirato J Kakita A Takahashi H Sugino T Tasaki K Itoh H Hatori T Shimoyama Y Analysis of Chromosome 19q13.42 Amplification in Embryonal Brain Tumors with Ependymoblastic Multilayered Rosettes Brain Pathol 2012 “Accepted Article”doi:10.1111/j.1750- 3639.2012.00574.x 10.1111/j.1750-3639.2012.00574.x PMC8057628 22324795

[B26] Fornari F Milazzo M Chieco P Negrini M Marasco E Capranico G Mantovani V Marinello J Sabbioni S Callegari E In hepatocellular carcinoma miR-519d is upregulated by p53 and DNA hypomethylation and targets CDKN1A/p21, PTEN, AKT3 and TIMP2 J Pathol 2012 "Accepted Article" 10.1002/path.3995 22262409

[B27] Augello C Vaira V Caruso L Destro A Maggioni M Park YN Montorsi M Santambrogio R Roncalli M Bosari S MicroRNA profiling of hepatocarcinogenesis identifies C19MC cluster as a novel prognostic biomarker in hepatocellular carcinoma Liver Int 2012 32 772 782 10.1111/j.1478-3231.2012.02795.x 22429613

[B28] Wolbank S Peterbauer A Fahrner M Hennerbichler S van Griensven M Stadler G Redl H Gabriel C Dose-dependent immunomodulatory effect of human stem cells from amniotic membrane: a comparison with human mesenchymal stem cells from adipose tissue Tissue Eng 2007 13 1173 1183 10.1089/ten.2006.0313 17518752

